# Favorable immune checkpoint inhibitor outcome of patients with melanoma and NSCLC harboring *FAT1* mutations

**DOI:** 10.1038/s41698-022-00292-6

**Published:** 2022-06-23

**Authors:** Wenjing Zhang, Yunfeng Tang, Yuxian Guo, Yujia Kong, Fuyan Shi, Chao Sheng, Suzhen Wang, Qinghua Wang

**Affiliations:** 1 Department of Health Statistics, Key Laboratory of Medicine and Health of Shandong Province, School of Public Health, Weifang Medical University, 261053 Weifang, Shandong China; 2 School of Public Health, Weifang Medical University, 261053 Weifang, Shandong China; 3 Department of Epidemiology and Biostatistics, National Clinical Research Center for Cancer, Key Laboratory of Molecular Cancer Epidemiology of Tianjin, Tianjin Medical University Cancer Institute and Hospital, 300060 Tianjin, China

**Keywords:** Cancer immunotherapy, Tumour immunology

## Abstract

Immune checkpoint inhibitors (ICIs) are most commonly used for melanoma and non-small cell lung cancer (NSCLC) patients. FAT atypical cadherin 1 (FAT1), which frequently mutates in melanoma and NSCLC. In this study, we aim to investigate the association of *FAT1* mutations with ICI response and outcome. We collected somatic mutation profiles and clinical information from ICI-treated 631 melanoma and 109 NSCLC samples, respectively. For validation, a pan-cancer cohort with 1661 patients in an immunotherapy setting was also used. Melanoma and NSCLC samples from the Cancer Genome Atlas were used to evaluate the potential immunologic mechanisms of *FAT1* mutations. In melanoma, patients with *FAT1* mutations had a significantly improved survival outcome than those wild-type patients (HR: 0.67, 95% CI: 0.46–0.97, *P* = 0.033). An elevated ICI response rate also appeared in *FAT1*-mutated patients (43.2% vs. 29.2%, *P* = 0.032). Associations of *FAT1* mutations with improved prognosis and ICI response were confirmed in NSCLC patients. In the pan-cancer cohort, the association between *FAT1* mutations and favorable ICI outcome was further validated (HR: 0.74, 95% CI: 0.58–0.96, *P* = 0.022). Genomic and immunologic analysis showed that a high mutational burden, increased infiltration of immune-response cells, decreased infiltration of immune-suppressive cells, interferon and cell cycle-related pathways were enriched in patients with *FAT1* mutations. Our study revealed that *FAT1* mutations were associated with better immunogenicity and ICI efficacy, which may be considered as a biomarker for selecting patients to receive immunotherapy.

## Introduction

Worldwide, melanoma causes ~56,000 deaths each year^1^. Because of distinct access to early diagnosis and timely treatment, the morbidity and mortality of melanoma differ widely by country. For a long time, few therapy strategies were used for melanoma clinical practice owing to the unsuccess of relevant clinical trials^2^. In recent years, the development of genomic sequencing technologies and deep exploration of biological mechanisms have changed melanoma into a novel treatment model. Targeted therapy (e.g., BRAF inhibitors) has been demonstrated to markedly improve patients’ response and clinical outcomes^3^. Moreover, immune checkpoint inhibitors (ICIs), which have a capacity to prolong survival outcomes of advanced or metastatic patients, have been become the routine clinical treatment pattern for melanoma^4,5^.

Non-small cell lung cancer (NSCLC) is the main histologic subtype of lung cancer. Several treatment strategies were reported for NSCLC. Metastatic NSCLC patients with EGFR mutations treated with EGFR tyrosine kinase inhibitors (TKIs) have exhibited an improved survival outcome^6^ and the EGFR-TKIs are standard drugs in first-line treatment^7^. Besides, the combination of VEGF inhibitors or other chemotherapies with EGFR-TKIs was also considered as a helpful therapeutic path for NSCLC^8^. The emergence of ICI therapies has dramatically lengthened the survival of NSCLC patients in advanced stage or patients who produced a treatment resistance during conventional chemotherapy^9^.

Although melanoma and NSCLC patients who received ICI agents have revealed a preferable prognosis; however, the fact is that in clinical practice only a minority of patients could obtain a treatment response to ICIs^10^. Recently multiple molecular markers were determined to select patients who are responsive or resistant to ICIs, for example, tumor mutation burden (TMB)^11,12^, neoantigen burden (NB)^13^, PD-L1 protein expression^14^, mutations in *POLE*^15^, *TP53*^16,17^, *MUC16*^18,19^, *PBRM1*^20^, and *B2M*^21^.

FAT atypical cadherin 1 (FAT1), which is a well-known tumor suppressor, plays tumor inhibition roles via the regulation of WNT/β-catenin signaling^22^, Hippo signaling^23^, and MAPK/ERK-signaling activities^24^. Loss of function of FAT1 contributed to tumor progression and impacted clinical outcomes. A recent study reported that FAT1 deletion generated a hybrid epithelial to mesenchymal transition status and thus promoted squamous cell carcinoma stemness and metastasis^25^. Consistent findings were observed in refs. ^26,27^ studies, that are, *FAT1* mutation was associated with an inferior survival outcome in head and neck squamous cell carcinoma (HNSCC). However, a previous research revealed an inverse conclusion that human papillomavirus (HPV)-negative HNSCC patients with *FAT1* mutation exhibited a better prognosis^28^. FAT1 loss was also demonstrated to be linked with the CDk4/6 inhibitor treatment resistance in breast cancer^29,30^. Besides, FAT1 or its alterations were involved in inflammatory regulation in glioma^31^ and clinically influenced T-cell lymphoma outcome^32^, suggesting that FAT1 may be an immune response regulator and participate in distinct inflammatory processes.

In this study, we retrospectively collected melanoma and NSCLC patients treated by ICIs to explore the association between *FAT1* mutations and immunotherapy efficacy. Furthermore, we conducted a series of genomic and immunologic analyses to illuminate the potential mechanisms behind *FAT1* mutations. Results from our work may provide helpful clues for enrolling cancer patients to receive immune treatments.

## Results

### *FAT1* mutations in melanoma

**Fig. 1:**
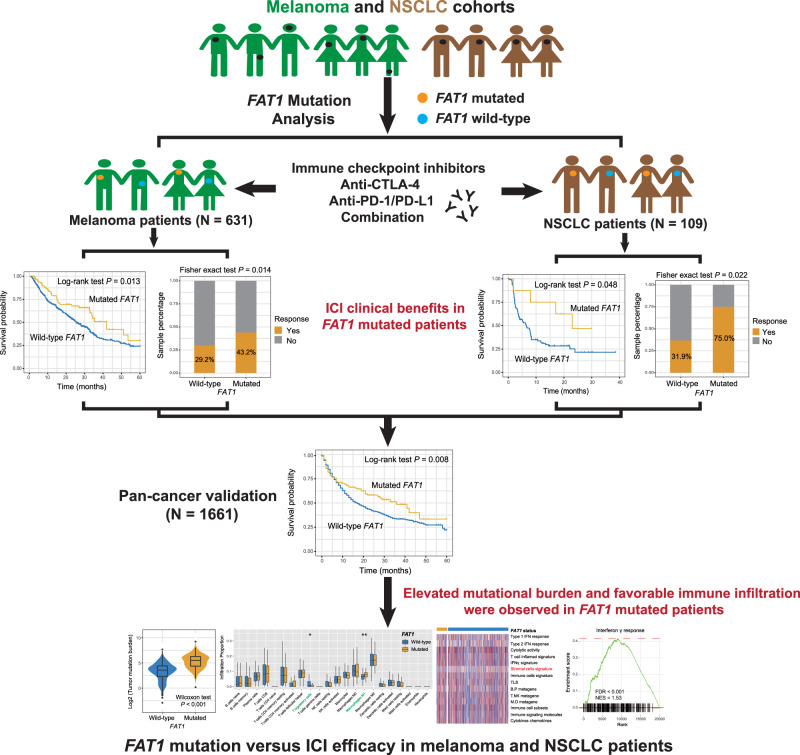
Detailed workflow of this study. *FAT1* mutations versus ICI efficacy in melanoma and NSCLC patients. Green samples, melanoma; brown samples, NSCLC; orange dot, mutated *FAT1*; blue dot, wild-type *FAT1*.

Of the 631 integrated melanoma samples, 193 (30.6%) exhibited the ICI status of complete response (CR) or partial response (PR), 430 (68.1%) were status of stable disease (SD) or progressive disease (PD), and the rest (1.3%) were unavailable. C>T was the main base substitution type in this melanoma cohort (Supplementary Fig. 1). Mutational patterns of *FAT1* and the most common significantly mutated genes were shown in Supplementary Fig. 1. We observed that *FAT1* is frequently mutated, accounting for 82 of 631 patients (12.9%). Amino acid transformations generated by *FAT1* mutations were illustrated using a lollipop plot (Supplementary Fig. 2). Detailed protein and function changes induced by *FAT1* mutations for each melanoma patient were shown in Supplementary Data 1. The workflow of this research was shown in Fig. 1.

### *FAT1* mutations linked with improved ICI efficacy in melanoma

**Fig. 2:**
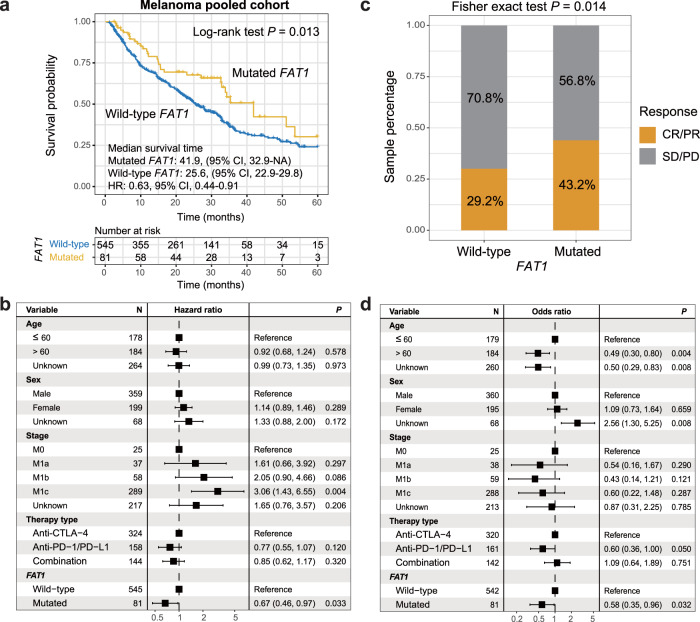
Association of *FAT1* mutations with ICI prognosis and response in melanoma cohort. **a** Kaplan–Meier survival curves stratified by *FAT1* mutational status. **b** Multivariate Cox regression model between *FAT1* mutations and ICI outcome with age, sex, stage, and therapy type taken into account. Black box: hazard ratio; black scale bar, 95% confidence interval of hazard ratio. **c** Distinct ICI response rates in *FAT1* mutated and wild-type subgroups. **d** Multivariate logistic regression model between *FAT1* mutations and ICI response with age, sex, stage, and therapy type taken into account.

In the pooled melanoma cohort, univariate survival analysis revealed that patients with *FAT1* mutations had a significantly improved ICI prognosis than those wild-type patients (median survival time: 41.9 vs. 25.6 months, Log-rank test *P* = 0.013; Fig. 2a). Multivariate Cox regression model with clinical confounding factors (e.g., age, sex, stage, and therapy type) taken into consideration still demonstrated a significant result (HR: 0.67, 95% CI: 0.46–0.97, *P* = 0.033; Fig. 2b). Associations between *FAT1* mutations and ICI prognosis in individual cohorts and distinct treatment types were, respectively, shown in Supplementary Figs. 3 and 4. Further analysis showed that *FAT1* mutated patients also exhibited an elevated ICI response rate (43.2% vs. 29.2%, Fisher exact test *P* = 0.014; Fig. 2c). And this result was still significant after adjusting for other confounding factors (OR: 0.58, 95% CI: 0.35–0.96, *P* = 0.032; Fig. 2d).

### Relationship between *FAT1* mutations and mutation burden in melanoma

**Fig. 3:**
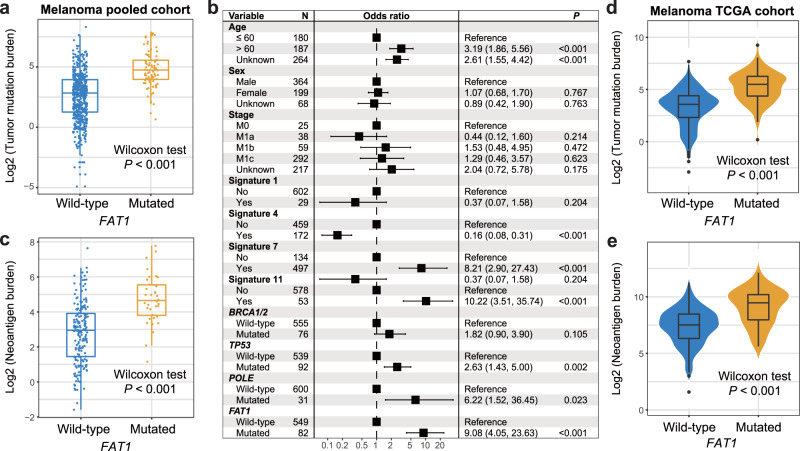
Association of *FAT1* mutations with mutation burden in melanoma. **a** Distinct distribution of TMB in *FAT1* mutated versus wild-type patients in the integrated melanoma cohort. Blue dot, TMB for wild-type patients; orange dot, TMB for mutated patients. **b** Multivariate logistic regression model between *FAT1* mutations and TMB with clinical confounding factors, extracted mutational signatures, and mutations in DNA repair genes taken into consideration. Black box: odds ratio; black scale bar, 95% confidence interval of odds ratio. **c** Distinct distribution of NB in *FAT1*-mutated versus wild-type patients in the integrated melanoma cohort. Distinct distribution of (**d**) TMB and (**e**) NB in *FAT1*-mutated versus wild-type melanoma patients from TCGA cohort.

We explored the relationship of *FAT1* mutations with TMB owing to its important roles in predicting cancer immune response and clinical outcome. We found that a significantly enhanced TMB was enriched in melanoma patients with *FAT1* mutations (Wilcoxon rank-sum test *P* < 0.001; Fig. 3a). Mutational signatures operative in the genome largely influence the genomic stability and mutation rates. We therefore extracted 4 mutational signatures from melanoma patients with the NMF method; they are signature 1 (age-relevant), signature 4 (smoking-relevant), signature 7 (ultraviolet light exposure-induced), and signature 11 (alkylating agent-induced). Activities of extracted signatures across all patients were illustrated in Supplementary Data 2. To eliminate the possibility that the association of *FAT1* mutations with TMB was impacted by other confounders, we combined clinical variables, extracted mutational signatures, and alterations in *BRCA1/2*, *TP53*, and *POLE* into the multivariate logistic regression. Association between *FAT1* mutations and higher TMB was still existed (OR: 9.08, 95% CI: 4.05–23.63, *P* < 0.001; Fig. 3b). Besides, *FAT1* mutations were also associated with a higher NB (Wilcoxon rank-sum test *P* < 0.001; Fig. 3c). Similar results of *FAT1* mutations with enhanced TMB and NB were also detected by using the melanoma somatic mutation data in the TCGA cohort (both *P* < 0.001; Fig. 3d, e).

### Validation in NSCLC

**Fig. 4:**
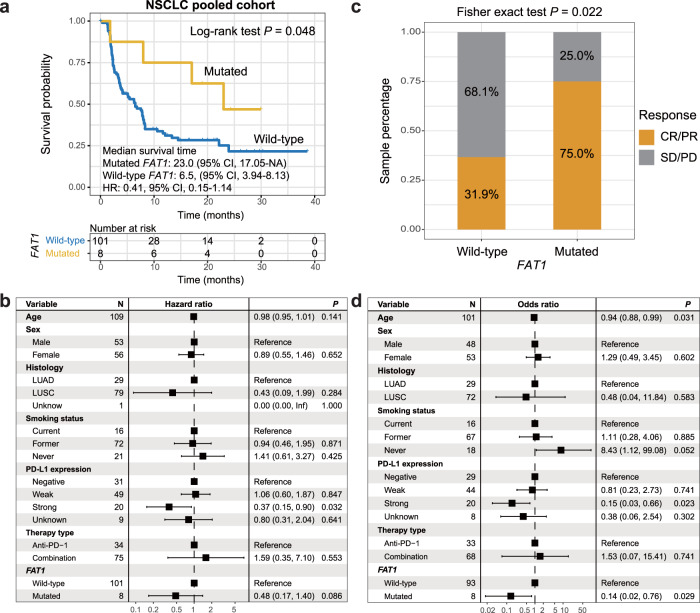
Association of *FAT1* mutations with ICI prognosis and response in NSCLC cohort. **a** Kaplan–Meier survival curves stratified by *FAT1* mutational status. **b** Multivariate Cox regression model between *FAT1* mutations and ICI outcome with age, sex, histology subtype, smoking status, PD-L1 expression, and therapy type taken into account. Black box: hazard ratio; black scale bar, 95% confidence interval of hazard ratio. **c** Distinct ICI response rates in *FAT1* mutated and wild-type subgroups. **d** Multivariate logistic regression model between *FAT1* mutations and ICI response with age, sex, histology subtype, smoking status, PD-L1 expression, and therapy type taken into account.

Of the 109 ICI-treated NSCLC patients, 36 (33.0%) harbored the status of CR or PR. *FAT1* mutated in 8 (7.3%) of 109 patients. Mutational patterns of *FAT1* and the most common NSCLC significantly mutated genes were shown in Supplementary Fig. 5. Detailed protein and function changes induced by *FAT1* mutations for each NSCLC patient were shown in Supplementary Data 3. Kaplan–Meier survival analysis showed that NSCLC patients with *FAT1* mutations had a significantly better survival outcome than wild-type patients (median survival time: 23.0 vs. 6.5 months, Log-rank test *P* = 0.048; Fig. 4a). Multivariate Cox regression model with confounding factors adjusted still revealed a consistent result, although it did not obtain a statistical significance (HR: 0.48, 95% CI: 0.17–1.40, *P* = 0.086; Fig. 4b). ICI prognostic capacity of *FAT1* mutations in distinct therapy types was illustrated in Supplementary Fig. 6. Furthermore, an elevated proportion of NSCLC patients with CR or PR was also observed in *FAT1* mutated subgroup (75.0% vs. 31.9%, Fisher exact test *P* = 0.022; Fig. 4c). This association was significant after controlling for multiple confounders (OR: 0.14, 95% CI: 0.02–0.76, *P* = 0.029; Fig. 4d).

**Fig. 5:**
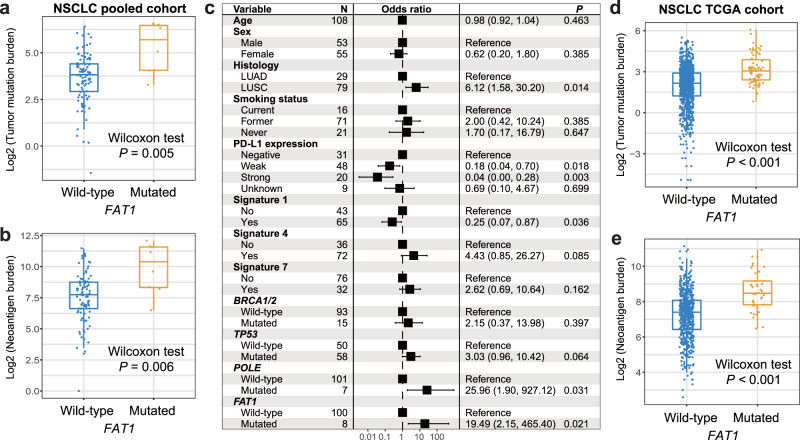
Association of *FAT1* mutations with mutation burden in NSCLC. **a** Distinct distribution of TMB in *FAT1* mutated versus wild-type patients in the integrated NSCLC cohort. Blue dot, TMB for wild-type patients; orange dot, TMB for mutated patients. **b** Multivariate logistic regression model between *FAT1* mutations and TMB with clinical confounding factors, extracted mutational signatures, and mutations in DNA repair genes taken into consideration. **c** Distinct distribution of NB in *FAT1* mutated versus wild-type patients in the integrated NSCLC cohort. Black box: odds ratio; black scale bar, 95% confidence interval of odds ratio. Distinct distribution of (**d**) TMB and (**e**) NB in *FAT1* mutated versus wild-type NSCLC patients from TCGA cohort.

Higher TMB and NB were observed in NSCLC patients with *FAT1* mutations (Wilcoxon rank-sum test *P* = 0.005 and 0.006, respectively; Fig. 5a, b). Three mutational signatures were extracted based on the mutational profile of NSCLC (Supplementary Data 4). Multivariate logistic analysis was conducted with age, sex, histology subtype, smoking status, PD-L1 expression, mutational signatures, and genome repair gene mutations taken into consideration; and the association of *FAT1* mutations with increased TMB was still obtained (OR: 19.49, 95% CI: 2.15–465.40, *P* = 0.021; Fig. 5c). We also noticed increased TMB and NB in *FAT1*-mutated subgroup in NSCLC patients from the TCGA (Wilcoxon rank-sum test, both *P* < 0.001; Fig. 5d, e).

### Further corroboration in a pan-cancer ICI cohort

**Fig. 6:**
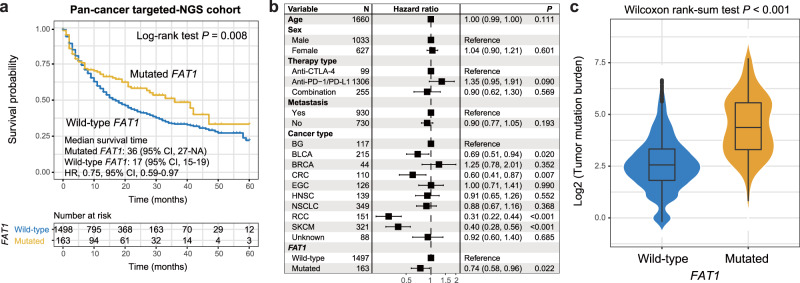
Validation of ICI predictive ability of *FAT1* mutations with a pan-cancer cohort. **a** Kaplan–Meier survival curves stratified by *FAT1* mutational status. **b** Multivariate Cox regression model between *FAT1* mutations and ICI outcome with clinical confounding factors taken into account. Black box: hazard ratio; black scale bar, 95% confidence interval of hazard ratio. **c** Association of *FAT1* mutations with TMB in the pan-cancer cohort.

By using a pan-cancer cohort with distinct cancer types from MSKCC, we further investigated the ICI predictive roles of *FAT1* mutations. Survival analysis showed that a significantly improved ICI prognosis was observed in *FAT1* mutated patients (median survival time: 36 vs. 17 months, Log-rank test *P* = 0.008; Fig. 6a). And multivariate Cox regression model suggested that *FAT1* mutation is an independent prognostic biomarker (HR: 0.74, 95% CI: 0.58–0.96, *P* = 0.022; Fig. 6b). In this cohort, an enhanced TMB was also noticed in patients with *FAT1* mutations (Wilcoxon rank-sum test *P* < 0.001; Fig. 6c).

### Immune infiltration and signaling pathways associated with *FAT1* mutations

**Fig. 7:**
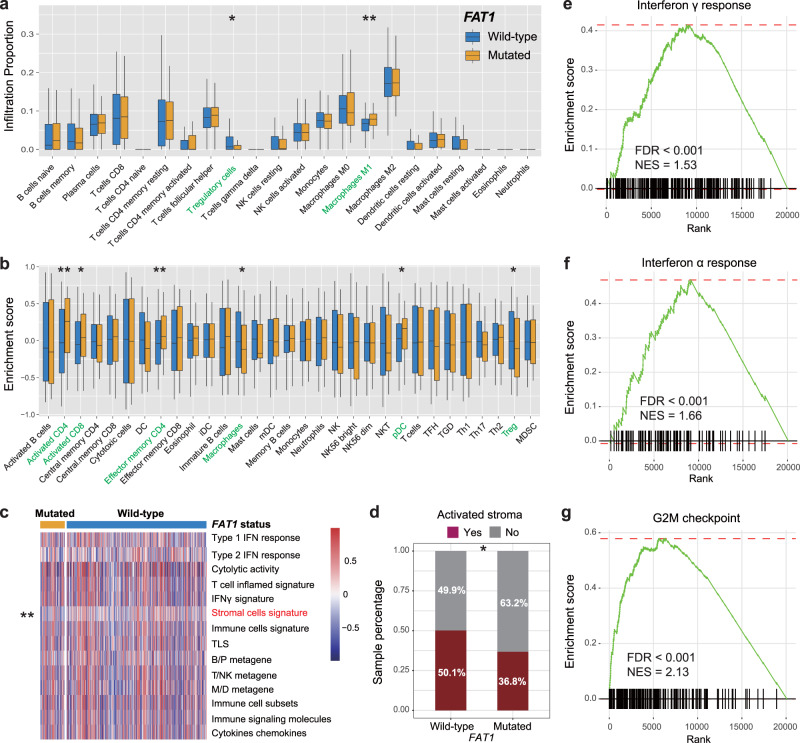
Evaluation of immune infiltration, immune signatures, and pathway enrichment in *FAT1*-mutated melanoma patients. Distinct immunocyte infiltration abundance in *FAT1* mutated versus wild-type subgroups was assessed with **a** CIBERSORT and **b** Angelova et al. methods, respectively. Blue box, *FAT1* wild-type patients; orange box, *FAT1*-mutated patients; black scale bar, range of each immunocyte infiltration abundance. Significantly differentially infiltrating immunocytes between two subgroups were highlighted with green. **c** Heatmap representation of immune-related signatures enrichment in distinct *FAT1* mutational subgroups. Significantly differentially enriched signature between two subgroups was highlighted with red. **d** Distinct proportion of activated-stroma subtype in distinct *FAT1* mutational subgroups. **e**–**g** Significantly enriched signaling pathways in *FAT1*-mutated melanoma patients. *P < 0.05, **P < 0.01.

We conducted multiple immunologic analyses and pathways exploration to illuminate the potential mechanisms behind *FAT1* mutations in melanoma. CIBERSORT algorithm revealed that increased infiltration of M1 macrophage and decreased infiltration of T-regulatory cells were enriched in *FAT1*-mutated subgroup (both *P* < 0.05; Fig. 7a). Consistently, by using Angelova et al. method, we observed *FAT1* mutations were positively associated with pro-inflammatory immunocyte (e.g., activated CD4/CD8 cells and effector memory CD4 cells) infiltration abundance; however, negatively associated with immune-suppressive T-regulatory cell infiltration (all *P* < 0.05; Fig. 7b).

We further composed a heatmap with distinct enrichment of immune-related signatures in *FAT1* mutated versus wild-type subgroups. Differential analysis demonstrated that the stromal cell signature exhibited a significantly reduced enrichment in *FAT1* mutated group (*P* < 0.001; Fig. 7c). Consistent with this finding, a lower proportion of patients with activated stroma was also found in this group (36.8% vs. 50.1%, Fisher exact test *P* < 0.05; Fig. 7d). GSEA analysis showed that immune response-related pathways (e.g., interferon α and γ responses) and cell cycle pathways (e.g., G2M checkpoint) were markedly enriched in melanoma patients with *FAT1* mutations (all FDR < 0.001; Fig. 7e–g, and Supplementary Fig. 7).

We also performed immune infiltration and pathway analyses in NSCLC patients. Results indicated that elevated infiltration of M1 macrophage and activated/effector memory CD4 cells, and decreased infiltration of M2 macrophage and T-regulatory cells were observed in *FAT1* mutation group (all *P* < 0.05; Supplementary Fig. 8a, b). Inflammatory and interferon response-related pathways were also noticed in this mutated group; however, the pathway of epithelial–mesenchymal-transition was enriched in *FAT1* wild-type subgroup (all FDR < 0.01; Supplementary Fig. 8c).

## Discussion

By using melanoma and NSCLC patients treated with ICIs, we retrospectively investigated the immunotherapy predictive roles of *FAT1* mutations. In addition, the association of *FAT1* mutations with favorable ICI outcome was observed in a pan-cancer cohort. Genomic and multiple immunological explorations further elucidated potential biological mechanisms underlying *FAT1* mutations. Findings obtained from this study suggest that *FAT1* mutation may be a potential indicator for assessing ICI efficacy.

In this work, we found that *FAT1* mutations were associated with an improved ICI prognosis. Further immune infiltration analysis showed an increased infiltrating abundance of CD4/CD8 T cells and a decreased abundance of T-regulatory cells in patients with *FAT1* mutations. CD4 and CD8 T cells are two well-known lymphocytes, which play a vital role in cancer immune response^33,34^. T-regulatory cells, which are a subtype of T cells, have been demonstrated to play an immune-suppressive function in anti-tumor immunity^35,36^. The dynamic interaction between tumor cells and their surrounding stroma influences the progression, metastasis, and drug resistance of cancer patients^37^. Recent studies have revealed that stromal cells inhibit the anti-tumor response and sensitivity to immunotherapy^38,39^. Yoshihara et al. developed the ESTIMATE method by using gene expression data to infer the stromal cell infiltration level in mixed tumor tissue^40^. Moffitt et al. determined a stroma-specific subtype via the utilization of stroma-related feature genes^41^. Based on the above two stroma evaluation methods, we observed *FAT1-*mutated patients had a reduced infiltration abundance of stromal cells and a reduced proportion of activated-stroma subtype. Interferon response pathways, which were enriched in *FAT1*-mutated subgroup, are positive immune response regulators^42,43^. Higher TMB and NB have been demonstrated as promising biomarkers for predicting prognosis and immunotherapy response in several cancers^11,12,44^, although there is still a controversy on TMB in some clinical settings^45,46^. In the present study, a markedly elevated TMB and MB was observed in *FAT1* mutated melanoma and NSCLC patients, which supports the potential ICI predictive roles of *FAT1* mutations. Further functional experiments and clinical trials are necessary to validate these findings.

FAT1 is a typical tumor suppressor and its determined mechanisms are involved in the WNT/β-catenin pathway^22^, Hippo pathway^23^, and MAPK/ERK pathways^24^. Mutations in *FAT1* always generate an inferior survival outcome in several cancers, such as NSCLC^25^ and HNSCC^26,27^. Recently, the immune regulation roles of FAT1 have been reported in multiple studies^31,32,47^. Dikshit et al. observed that FAT1-mediated glioma inflammation response via regulating the activity of PDCD4 and transcription factor AP-1^31^. In peripheral T-cell lymphoma, *FAT1* was frequently mutated and accounted for a significant proportion (39%) of patients, which provided both prognostic and therapeutic implications^32^. Grandi et al. reported that vaccination with a combination of FAT1-derived B cell epitope with tumor-specific B and T cell epitopes conferred a robust protective role in cancer mouse models^47^. The above findings further confirm the crucial roles of FAT1 in immune regulation and provide evidence for the present study.

Association of *FAT1* mutations with drug sensitivity was recently reported^29^, that is, *FAT1* loss-of-function mutations were associated with a resistance to CDK4/6 inhibitors in estrogen receptor-positive breast cancer. We also evaluated the prognostic roles of *FAT1* mutations in melanoma and NSCLC patients treated with distinct chemotherapies from TCGA cohorts; and no significant survival differences were observed between *FAT1* mutated and wild-type subgroups in both tumors (Log-rank test, both *P* > 0.05; Supplementary Fig. 9). In this study, we observed that patients with *FAT1* mutations exhibited a favorable prognosis in melanoma, NSCLC, and pan-cancer cohorts with immunotherapy settings. These results indicate that *FAT1* mutations play distinct roles in distinct treatment environments and it may be a potential predictive biomarker in the settings of cancer immunotherapy.

We further explored whether *FAT1* mutations affect ICI treatment efficacy by regulating *FAT1* expression. By using somatic mutation and transcriptomic data from TCGA melanoma and NSCLC cohorts, we analyzed the distinct *FAT1* expressions in patients with distinct *FAT1* mutational types (Supplementary Figs. 10 and 11). Results showed no significant expression differences were observed in all comparison subgroups (Wilcoxon test, all *P* > 0.05), which suggests that *FAT1* mutation may not influence its own expression levels in melanoma and NSCLC. Further in-depth studies regarding the association of *FAT1* mutations with *FAT1* expression are warranted.

In this study, we curated eight independent melanoma datasets into a pooled cohort and observed that *FAT1* mutations were associated with a favorable ICI response and outcome. Nevertheless, in individual cohorts and distinct ICI treatment types, *FAT1* mutations sometimes lacked the predictive ability of immunotherapy efficacy (Supplementary Figs. 3 and 4), the relatively smaller sample size for each cohort may be a possible reason. Therefore, clinically expanded cohorts with data integration are necessary to robustly determine immunotherapy efficacy-related indicators^48,49^.

Limitations exist in this study. First, the association between *FAT1* mutations and ICI efficacy is derived from previously published datasets, which is a retrospective study. Therefore, melanoma and NSCLC cohorts with both somatic mutation data and immunotherapy information in a prospective design are necessary. Second, this integrated study comprises several distinct cohorts, which may produce some biases in data analysis. Moreover, the lack of experimental validation is another limitation.

In summary, by integrating genomic profiles and clinical ICI data, we identified that *FAT1* mutations were predictive of ICI response and outcome in melanoma, NSCLC, and pan-cancers. Further in-depth studies are needed, but *FAT1* mutations may be a novel selection for enrolling cancer patients to receive immunotherapies.

## Methods

### Sample collection

A total of 631 melanoma samples with both somatic mutation profiles (generated by whole-exome sequencing [WES]) and clinical information were collected from previous 8 studies^48,50–56^. Besides, 109 NSCLC samples were collected from recent two studies^13,57^. In the above included studies, melanoma and NSCLC patients were treated with anti-PD1/PD-L1, anti-CTLA-4, or combination treatments. The Oncotator was employed to uniformly annotate somatic mutations for all patients^58^. Non-synonymous mutations were taken into consideration to conduct relevant analyses. Detailed treatment response information, sequencing platforms, and demographic features were shown in Supplementary Data 5 for melanoma and Supplementary Data 6 for NSCLC.

A pan-cancer ICI cohort of 1661 patients with 9 diverse cancer types in the Memorial Sloan Kettering Cancer Center (MSKCC) was also curated for further verification^11^. These patients underwent a 468-gene targeted sequencing and their clinical information was illustrated in Supplementary Data 7.

From Genome Data Commons (https://gdc.cancer.gov), we downloaded transcriptomic data, somatic mutational data, and clinical features of 457 melanoma and 995 NSCLC samples in the Cancer Genome Atlas (TCGA) cohort. The potential mechanistic analyses were performed based on the transcriptomic data of TCGA. All samples included in this study were acquired from previously published studies and the corresponding Institutional Ethics Committees have approved the studies.

### Mutational signature extraction

We used the method proposed by Kim et al. ^59^ to extract mutational signatures from aggregated melanoma and NSCLC samples. In this method, Bayesian variant nonnegative matrix factorization (NMF) was applied to decompose mutation portrait matrix *A* with 96 base substitution categories into two nonnegative matrices *W* and *H* (i.e., *A* ≈ *W* × *H*), where *W* indicating the extracted mutational signatures and *H* representing the mutational activities of each mutational signature. All determined mutational signatures were then compared with the 30 well-annotated signatures in the COSMIC database (version 2) based on cosine similarity.

### Tumor infiltrating immunocyte estimation

CIBERSORT method was used to evaluate the tumor abundance of 22 immunocyte subtypes with 547 feature genes from the LM22 signature^60^. Angelova et al. developed an 812-immune-metagene model to assess the infiltrating abundance of 31 immune cells^61^, gene panels for each immunocyte type were shown in Supplementary Data 8. In this study, we employed both methods to obtain a comprehensive result.

### Collection of immune-related signatures

Recently revealed representative immune-related signatures were curated as follows: (1) immune and stromal cells signatures, which suggest the total immune and stromal cell infiltration abundance in the microenvironment^40^; (2) immune cell subsets, evaluation of T cells, B cells, and NK cells infiltration^62^; (3) T/NK, B/P, and M/D metagene, which, respectively, suggests the enrichment of T/NK cells, B/plasma cells, and monocytes/dendritic cells^63^; (4) Type 1/2 IFN response, which are two interferon responses featured by interferon α and γ, respectively^64^; (5) IFNγ signature, which is a well-known indicator for immune response and ICI outcome^16^; (6) T cell-inflamed signature, an indicator derived from the IFNγ signature^65^; (7) immune cytolytic activity^64^; (8) immune signaling molecules^62^; (9) cytokines and chemokines^62^; (10) TLS, which is tertiary lymphoid structures and links with immune response^66^. Detailed feature genes for each immune signature were illustrated in Supplementary Data 9.

### Activated-stroma signature

A stroma-relevant signature^41^, which was characterized by two features (i.e., activated-stroma and normal-stroma) was proposed by Moffitt et al. study. By using the nearest template prediction (NTP) method^67^ with distinct feature genes of two stroma subgroups, we could obtain an activated stromal subtype.

### GSVA and GSEA

Single sample gene set enrichment analysis (ssGSEA) method in the GSVA package^36^ was employed to calculate enrichment scores of collected immune signatures for each sample with distinct gene sets. Gene expression differential analysis according to *FAT1* status was performed with R package DESeq2^37^. The obtained *t* values were subsequently regarded as the input to conducting gene set enrichment analysis (GSEA) in the fgsea package (https://github.com/ctlab/fgsea). Signaling pathways from the Hallmark and KEGG databases were utilized as the background circuits.

### Acquisition of mutational burden

Tumor mutation burden (TMB) was defined as the log2 transformation of total non-synonymous mutations per megabase in both WES and TCGA cohorts; for the MSKCC cohort, TMB was obtained from the supplementary information. The neoantigen burden (NB) of 224 melanoma and 109 NSCLC WES samples was estimated based on the method reported by Balachandran et al. ^68^. The neoantigen data of 340 melanoma and 656 NSCLC samples in the TCGA cohort was acquired from the Cancer Immunome Atlas (TCIA, https://www.tcia.at/home) project.

### Statistical analyses

R software (version 4.0.5) was employed to achieve related analyses. Gene mutational patterns were shown with maftools package^69^. Heatmap illustration of two subgroups was completed with pheatmap package. Kaplan–Meier method was used to produce survival curves and the Log-rank test to compare the differences. Multivariate regression models within forestmodel package were utilized to adjust confounding factors. Relationship of continuous and categorical variables with *FAT1* status was calculated with Wilcoxon rank-sum test and Fisher exact test, respectively.

### Reporting summary

Further information on research design is available in the Nature Research Reporting Summary linked to this article.

## Supplementary information

Supplementary Data

Supplementary Figures

REPORTING SUMMARY

## Data Availability

All data used in this study were acquired from publicly available cohorts, which are described in the “Methods” section.

## Code availability

All codes supported the results of this study are available from the corresponding author upon reasonable request.
