# Supplementary material for: Favorable immune checkpoint inhibitor outcome of patients with melanoma and NSCLC harboring FAT1 mutations
Source: NPJ Precis Oncol. 2022 Jun 23;6:46. doi: 10.1038/s41698-022-00292-6 (PMC9226130; doi:10.1038/s41698-022-00292-6)
Supplement: Supplementary file 2 — Supplementary Figures [file 41698_2022_292_MOESM2_ESM.docx]

**Supplementary Figures**

**
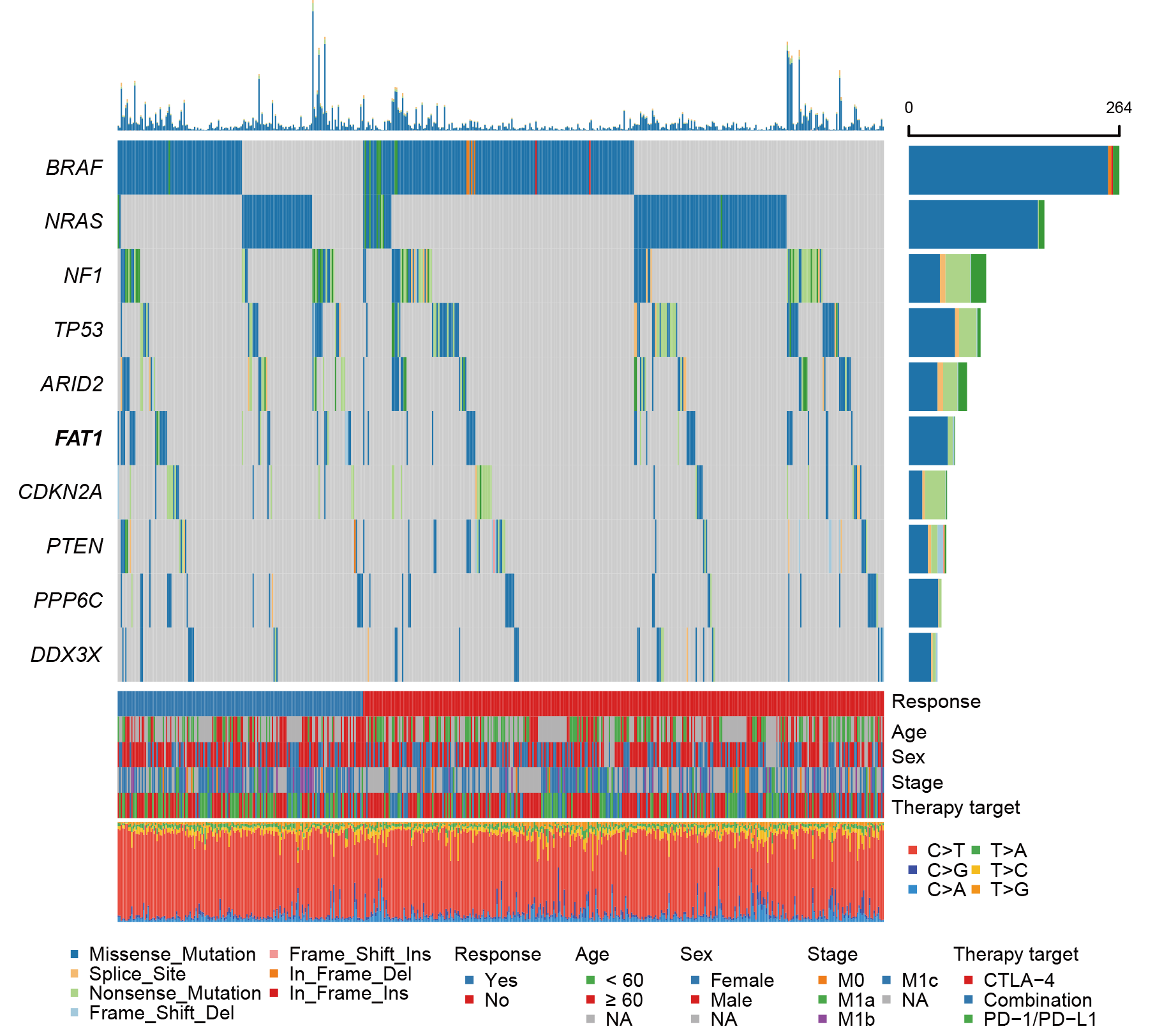
****Supplementary Figure 1. Mutational patterns of *FAT1* and commonly significantly mutated genes in the integrated melanoma cohort.** The left panel shows the gene symbols, the upper panel indicates the non-synonymous mutation counts for each melanoma patients, the middle plot illustrates SMGs mutational patterns with different mutation types colored differently, the right penal shows the mutation rate of each SMG, and the bottom panel indicates clinical characteristics and base substitution categories.

**
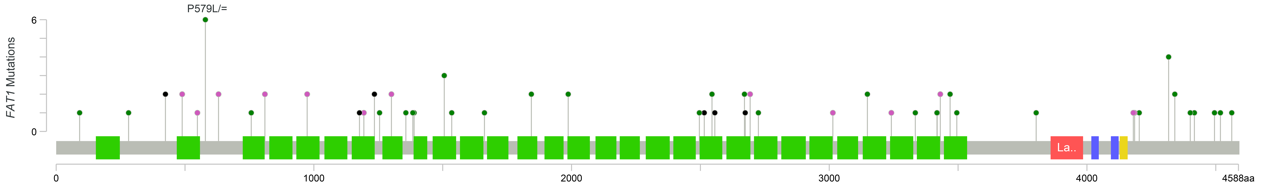
**

**Supplementary Figure 2. The detailed amino acid changes generated by *FAT1* mutations in melanoma cohort.** X-axis, amino acid; Y-axis, numbers of *FAT1* mutations; green dot, missense mutation; black dot, truncating mutation; pink dot, frameshift mutation.

**
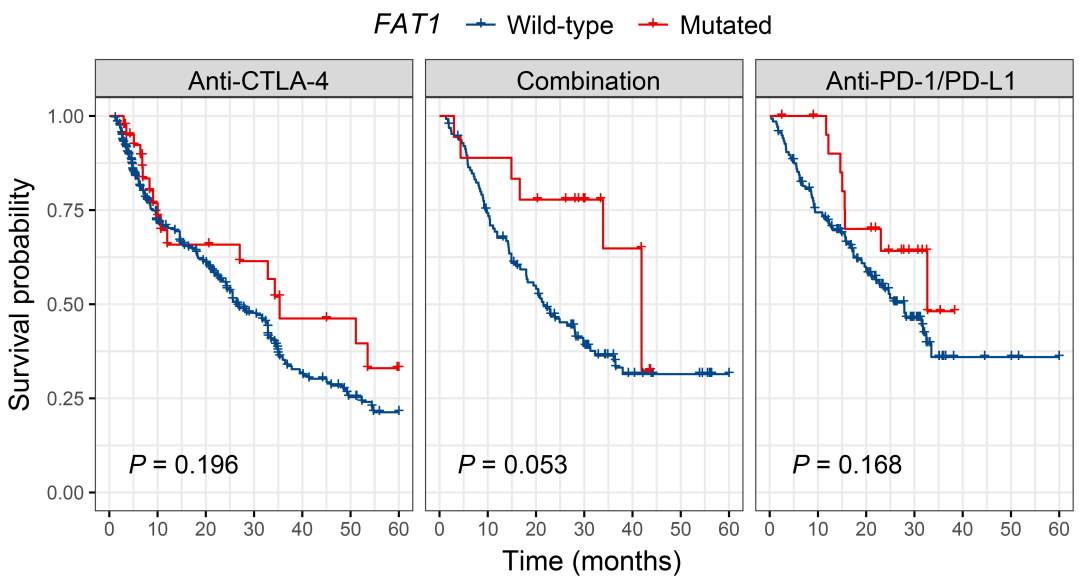

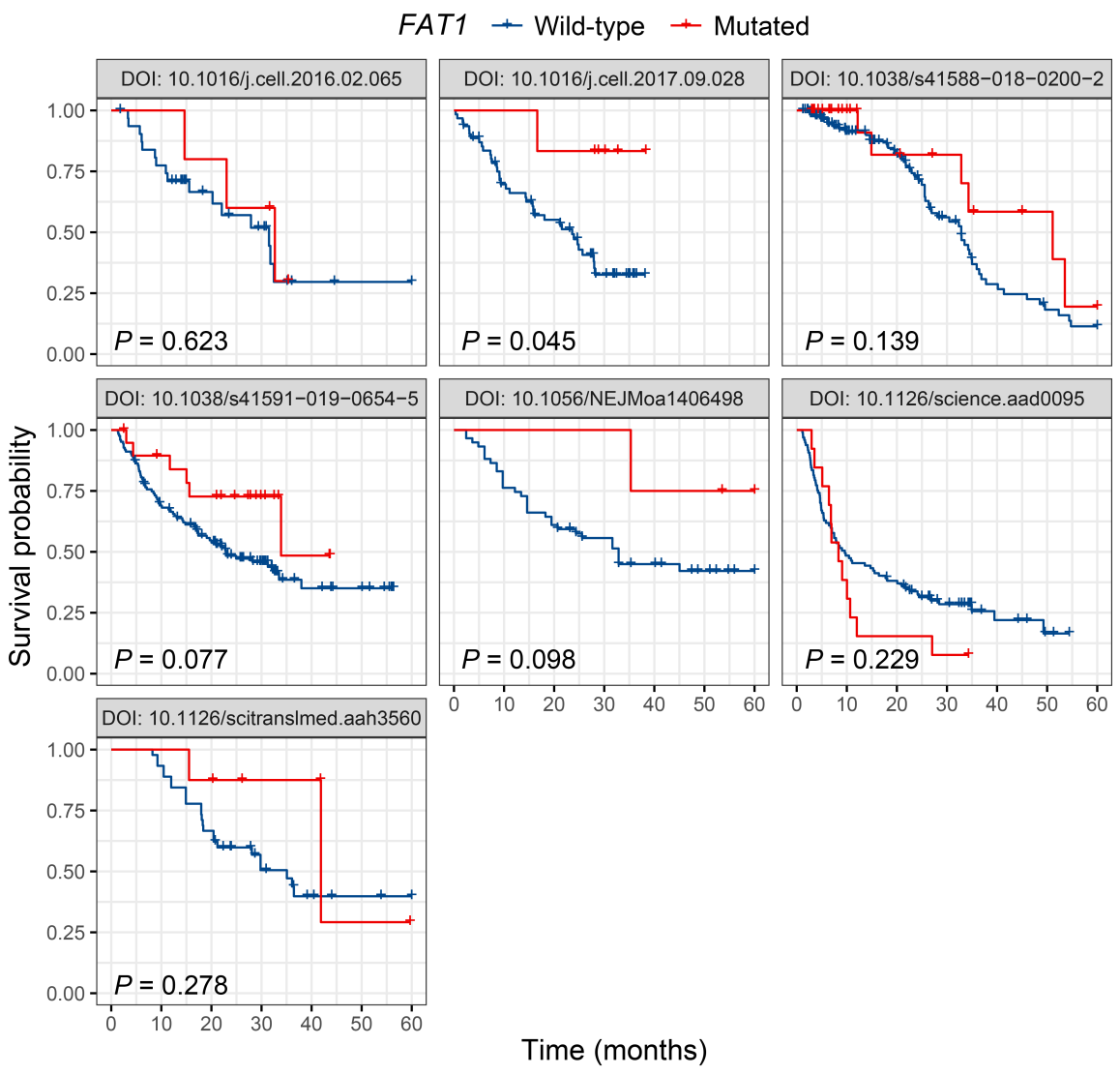
Supplementary Figure 3. Associations of *FAT1* mutations with survival outcomes in individual melanoma immunotherapy cohort.** Red line: mutated *FAT1*; blue line: wild-type *FAT1*.

**Supplementary Figure 4. Associations of *FAT1* mutations with survival outcomes in melanoma patients treated with distinct immunotherapy types.** Red line: mutated *FAT1*; blue line: wild-type *FAT1*.

**
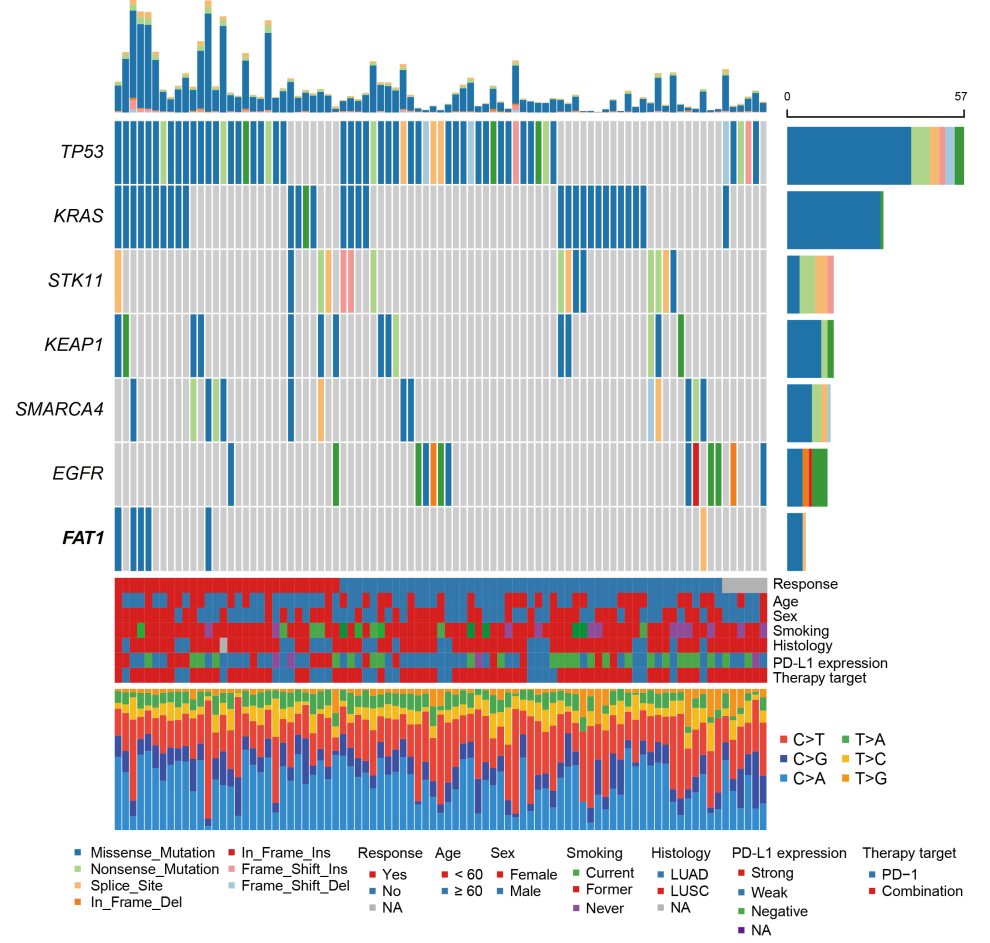
Supplementary Figure 5. Mutational patterns of *FAT1* and commonly significantly mutated genes in the integrated NSCLC cohort.** The left panel shows the gene symbols, the upper panel indicates the non-synonymous mutation counts for each NSCLC patients, the middle plot illustrates SMGs mutational patterns with different mutation types colored differently, the right penal shows the mutation rate of each SMG, and the bottom panel indicates clinical characteristics and base substitution categories.

**Supplementary Figure
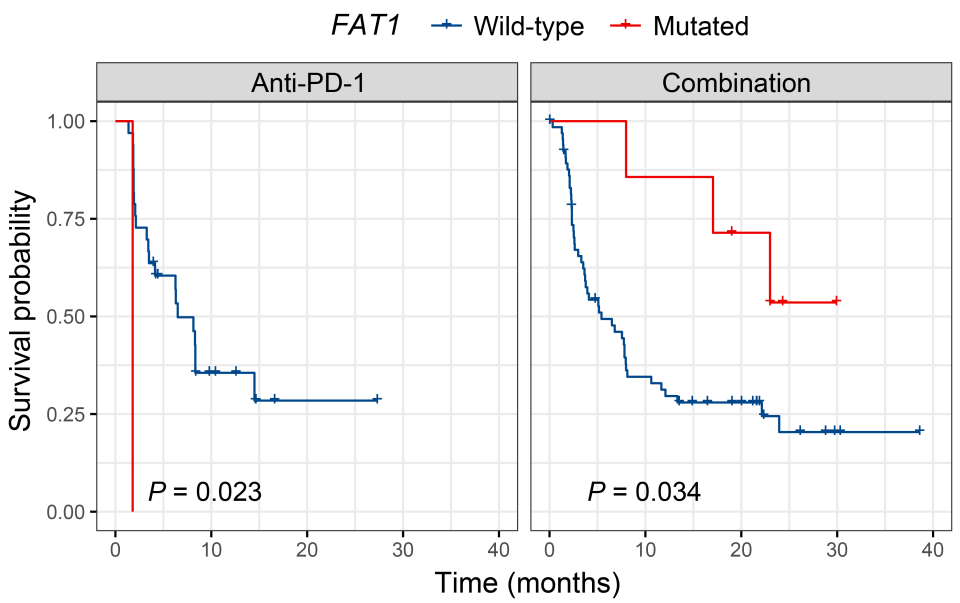
6. Associations of *FAT1* mutations with survival outcomes in NSCLC patients treated with distinct immunotherapy types.** Red line: mutated *FAT1*; blue line: wild-type *FAT1*.

**
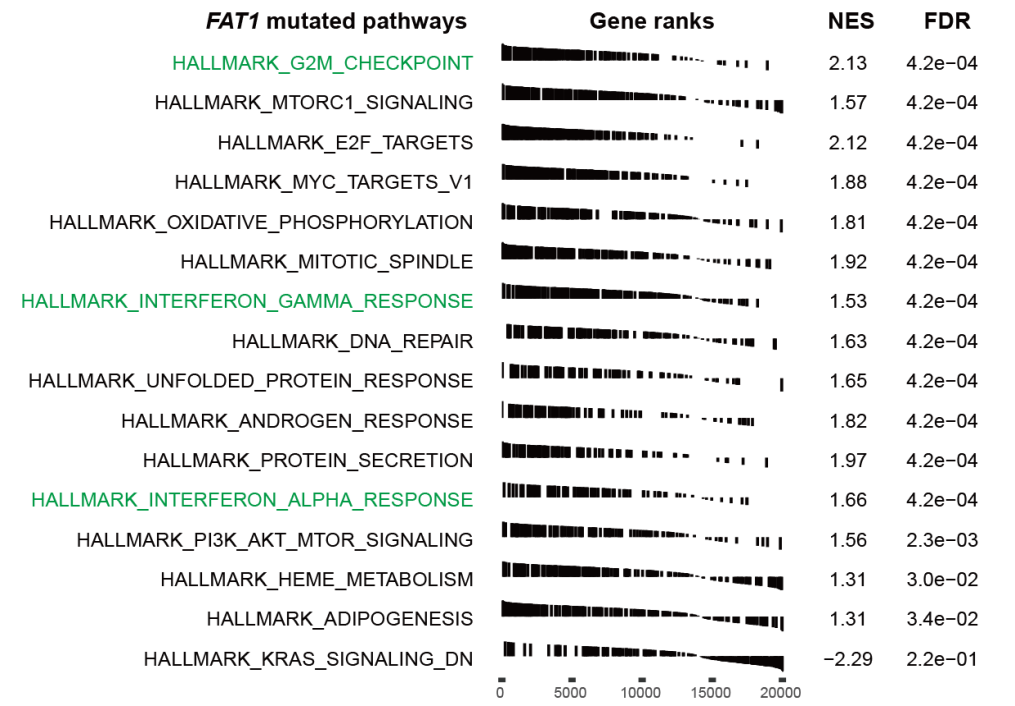
Supplementary Figure 7. Significantly enriched signaling pathways in *FAT1* mutated melanoma patients.** Green pathways, immune response-related; NES, normalized enrichment score; FDR, false discovery rate.

**
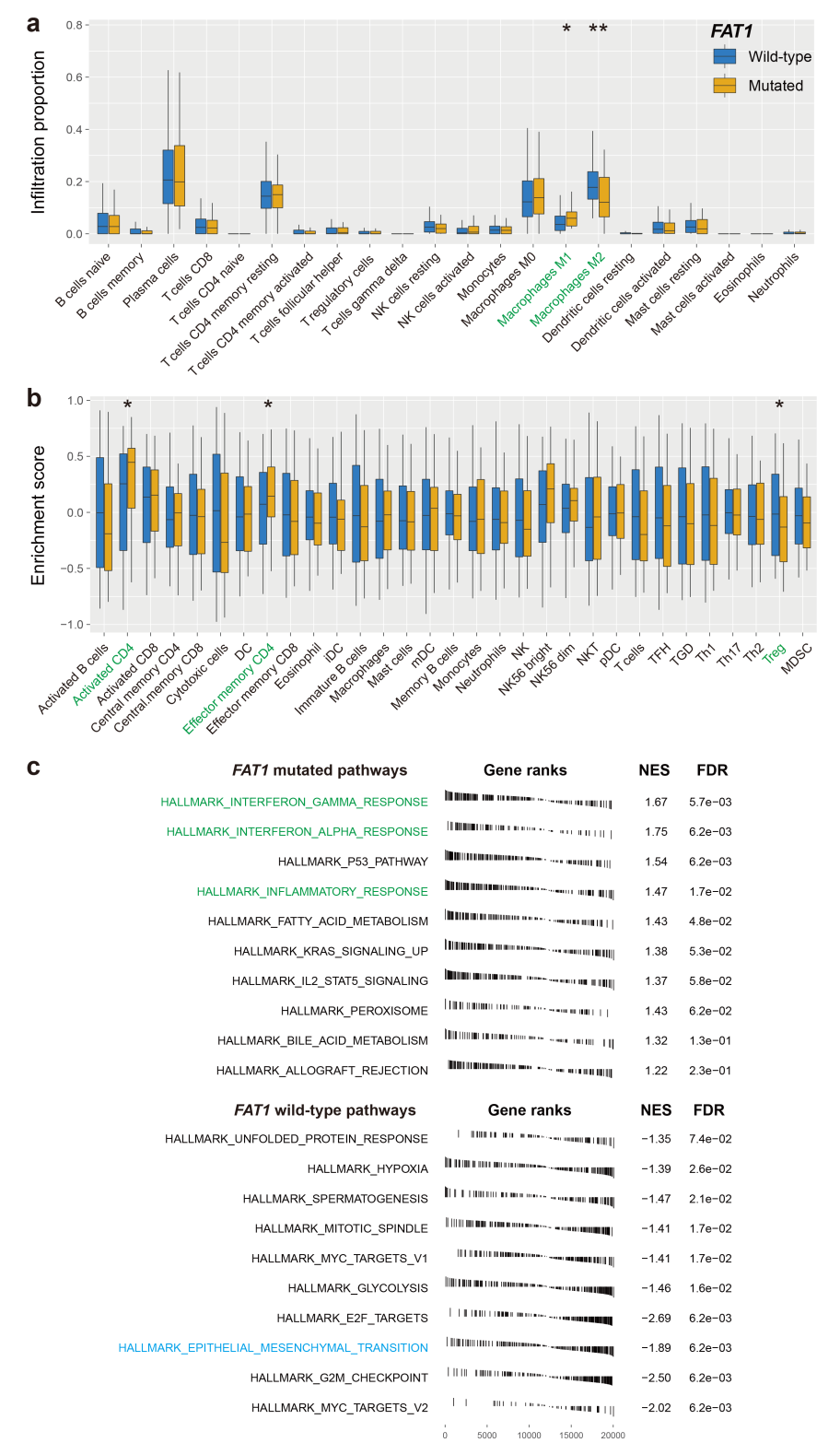
**

**Supplementary Figure 8. Immunocyte infiltration and GSEA analyses of *FAT1* mutations in NSCLC.** (A) Distinct immunocyte infiltration abundance evaluated by CIBERSORT algorithm in *FAT1* mutated and wild-type subgroups. Significantly differentially infiltrating immunocytes were highlighted with green. (B) Distinct immunocyte infiltration abundance evaluated by Angelova *et al*. method in *FAT1* mutated and wild-type subgroups. (C) Significantly enriched signaling pathways in *FAT1* mutated and wild-type patients. Immune response-related pathways were highlighted with green, while the immune suppressive pathway was blue.

**
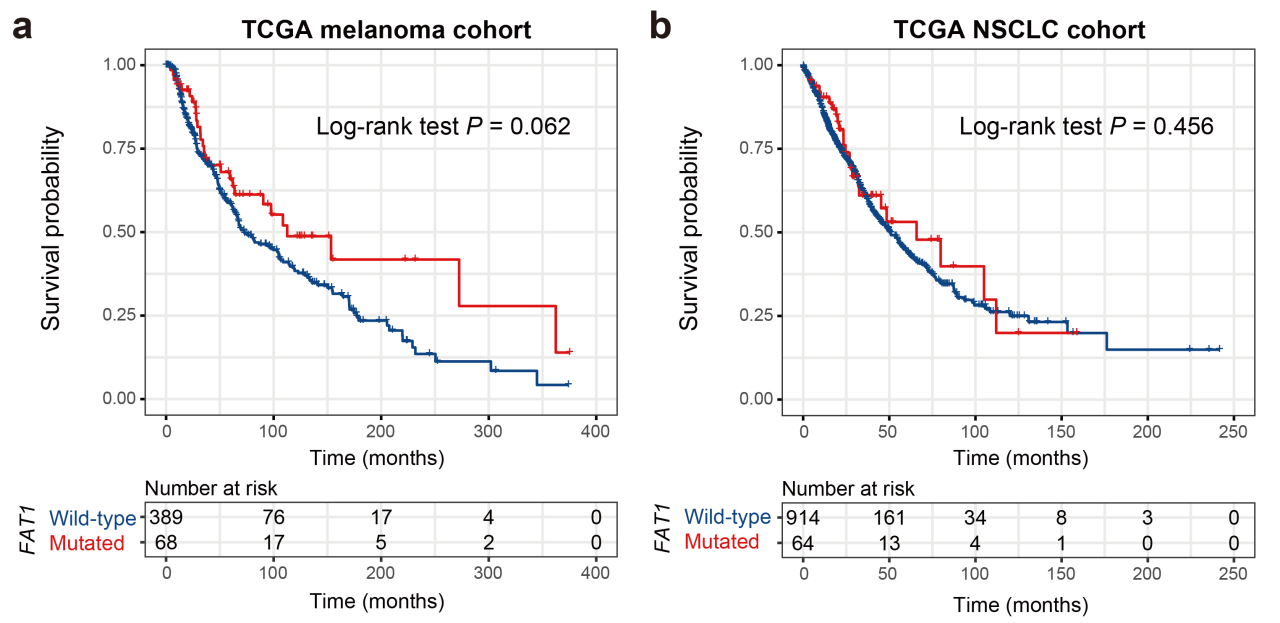
Supplementary Figure 9. Associations of *FAT1* mutations with survival outcomes in (A) melanoma and (B) NSCLC patients from TCGA cohorts.** Red line: mutated *FAT1*; blue line: wild-type *FAT1*.

**
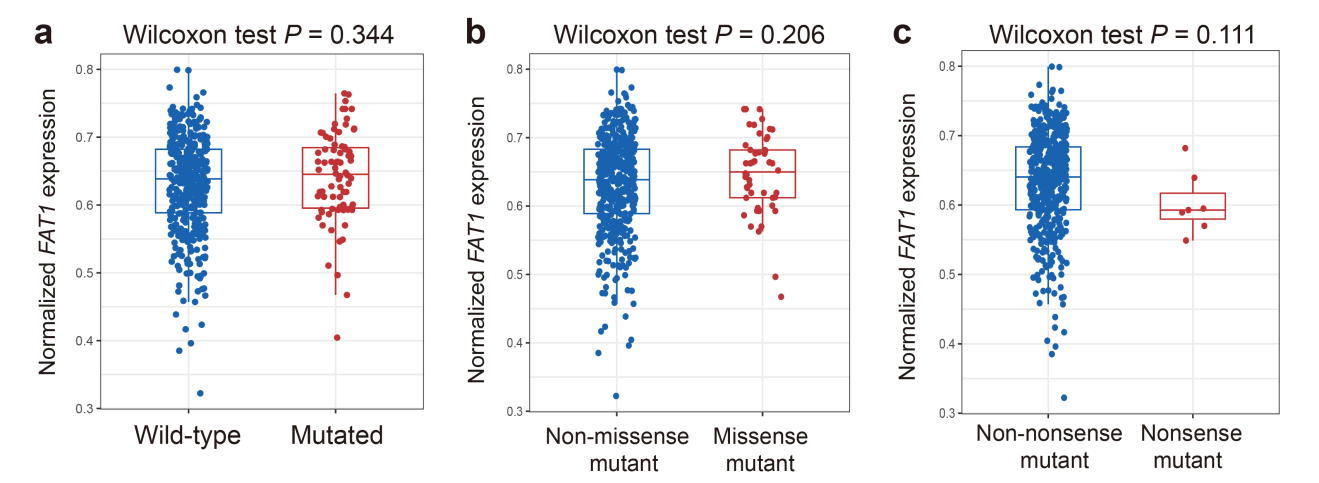
**

**Supplementary Figure 10. Distinct *FAT1* expressions in melanoma patients with distinct *FAT1* mutational types.** *FAT1* expression distribution in (A) *FAT1* mutant versus wild-type subgroups, (B) missense mutant versus non-missense mutant subgroups, and (C) nonsense mutant versus non-nonsense mutant subgroups. Blue and red dots represent *FAT1* wild-type and mutated patients, respectively, box plots indicate the median *FAT1* expression level and its interquartile ranges.

**
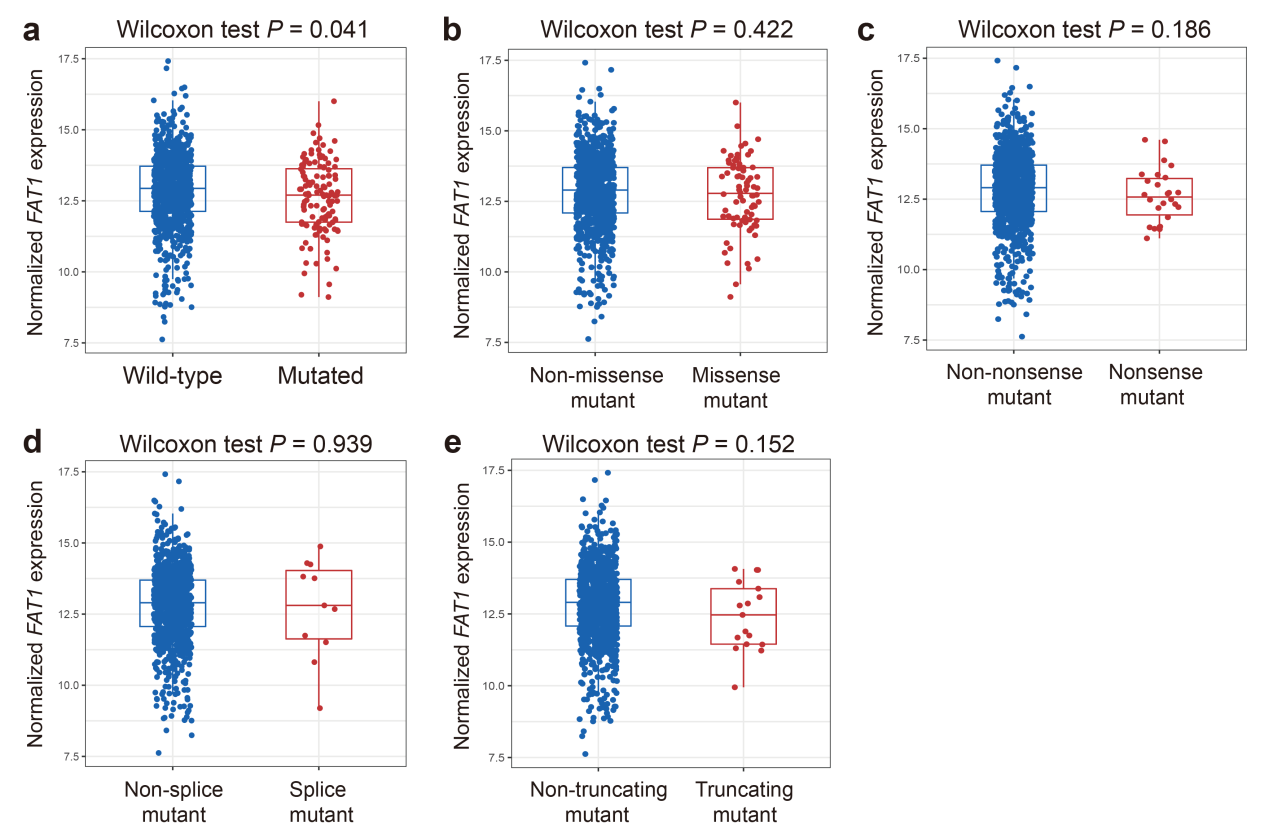
**

**Supplementary Figure 11. Distinct *FAT1* expressions in NSCLC patients with distinct *FAT1* mutational types.** *FAT1* expression distribution in (A) *FAT1* mutant versus wild-type subgroups, (B) missense mutant versus non-missense mutant subgroups, (C) nonsense mutant versus non-nonsense mutant subgroups, (D) splice mutant versus non-splice mutant subgroups, and (E) truncating mutant versus non-truncating mutant subgroups. Blue and red dots represent *FAT1* wild-type and mutated patients, respectively, box plots indicate the median *FAT1* expression level and its interquartile ranges.
